# Comorbidity patterns and implications for disease control: a network analysis of medical records from Shanghai, China

**DOI:** 10.3389/fpubh.2025.1516215

**Published:** 2025-03-10

**Authors:** Yifei Shen, Wenqi Tian, Na Li, Yuhong Niu

**Affiliations:** ^1^Shanghai Health Development Research Center (Shanghai Medical Information Center), Shanghai, China; ^2^Shanghai Health Statistics Center, Shanghai, China

**Keywords:** older adults, aging, chronic disease, comorbidity network, community classification, disease prevention

## Abstract

**Background:**

The aging problem in Shanghai is rapidly increasing, leading to the development of chronic comorbidities in older adults. Studying the correlations within comorbidity patterns can assist in managing disease prevention and implicate early control.

**Objectives:**

This study was a cross-sectional analysis based on a large sample size of 3,779,756 medical records. A network analysis and community classification were performed to illustrate disease networks and the internal relationships within comorbidity patterns among older adults in Shanghai.

**Methods:**

The network analysis and community classification were performed using the IsingFit and Fast-greedy community functions. Datasets, including disease codes and disease prevalence, were collected from medical records.

**Results:**

The top five prevalent diseases were hypertension (64.78%), chronic ischemic heart disease (39.06%), type 2 diabetes mellitus (24.97%), lipid metabolism disorders (21.79%), and gastritis (19.71%). The sampled population showed susceptibility to 11 comorbidities associated with hypertension, 9 with diabetes, 28 with ischemic heart disease, 26 with gastritis, and 2 with lipid metabolism disorders in male patients. Diseases such as lipid metabolism disorders, gastritis, fatty liver, polyps of the colon, osteoporosis, atherosclerosis, and heart failure exhibited strong centrality.

**Conclusion:**

The most common comorbidity patterns were dominated by ischemic heart disease and gastritis, followed by a ternary pattern between hypertension, diabetes, and lipid metabolism disorders. Male patients were more likely to have comorbidities related to cardiovascular and sleep problems, while women were more likely to have comorbidities related to thyroid disease, inflammatory conditions, and hyperuricemia. It was suggested that healthcare professionals focus on monitoring relevant vital signs and mental health according to the specific comorbidity patterns in older adults with chronic diseases, to prevent the development of new or more severe comorbidities.

## 1 Introduction

The World Health Organization (WHO) defines comorbidity as the presence of two or more persistent health conditions in the same individual, requiring prolonged intervention for maintenance (1). By the middle of this century, it is projected that the population aged 60 years and above in China will reach 430 million (2). Chronic comorbidity has long been a significant health challenge among older adults. In Shanghai, one of the regions undergoing severe aging problems, the detection rate of chronic diseases among older adults has reached 70% (3). Compared to single-diagnosed diseases, comorbidity poses a more substantial threat to health, as the interplay of various diseases creates a complex network of risk factors, further increasing mortality risk among patients (4). The growing trend of comorbidity not only complicates diagnosis and treatment but also places a significant economic burden on families as well as society. Therefore, there is an urgent need for effective strategies to prevent and manage comorbidities, as well as to provide professional guidance for caregiving to older adults and their families.

Several domestic and international studies have conducted in-depth research on the prevalence of comorbidity and the associations between different chronic conditions among older adults in various regions, utilizing data collected from epidemiological surveys and medical records, combined with the practical application of various network theories. These studies also provided recommendations for control approaches of different comorbidity patterns. A study applied a self-organizing map (SOM) neural network to visually present the associations between various common chronic diseases among middle-aged and older adults in China. They explored the differences in comorbidity patterns affected by age, gender, urban–rural residence, and treatment outcomes through visual cluster analysis (5). A study used a network mapping strategy to explore comorbidity patterns among the Zhuang population in Guangxi, identifying nine patterns strongly associated with hypertension. These findings helped improve the health condition of the study population (6). Another study examined comorbidity networks in communities within Jiangsu Province based on large-scale network analysis tools to figure out comorbidity patterns, disease prevalence, clustering characteristics, risk factors, and preventive strategies (7). In addition, a South Korean study focused on comorbidity patterns among obese populations demonstrated the relationship between several chronic diseases and obesity across different genders (8). However, there were still some limitations to the current research progress. Previous studies had been limited to a few common diseases and had primarily focused on specific regions, lacking normality across the whole target population in China. Although some studies had collected data from whole areas within the country, the dataset required an update.

Currently, the situation regarding comorbidity among older adults in Shanghai remains unclear. This study applied visual network approaches to medical records from outpatient, emergency, and hospital visits among individuals aged 60 to 99 years in Shanghai, aiming to illustrate the coexistent diseases associated with the top five most prevalent conditions: hypertension, chronic ischaemic heart disease, type 2 diabetes mellitus, gastritis, and lipoprotein metabolism disorders. The study focused on analyzing potential comorbidities of these five diseases and aimed to provide insights into the prevention and management of chronic comorbidity among older adults in Shanghai. Additionally, it might lay a solid foundation for future exploration of potential associations and control of chronic disease incidence.

## 2 Materials and methods

### 2.1 Data processing and study population

The dataset was collected from the Shanghai Municipal Health Commission database (9). It was derived from a collection of medical records of outpatient visits, emergency visits, and hospitalized records from a total number of 520 public, private, and community hospitals, based on the enrollment of country’s national basic medical insurance programs. Medical visits were traced by ID number enrolled in the insurance system during July 2022 and June 2023. The accuracy of the medical records uploaded was inspected under rigorous quality control. Repeat visits under one ID for the same disease were only counted once and combined into one record. In this case, each line of record represented the occurrence of all diseases for a single patient without duplication, no matter how many times patients repeat their visits for the same disease. In this study, we defined that the number of visiting records represented the prevalence of each disease.

**Figure 1:**
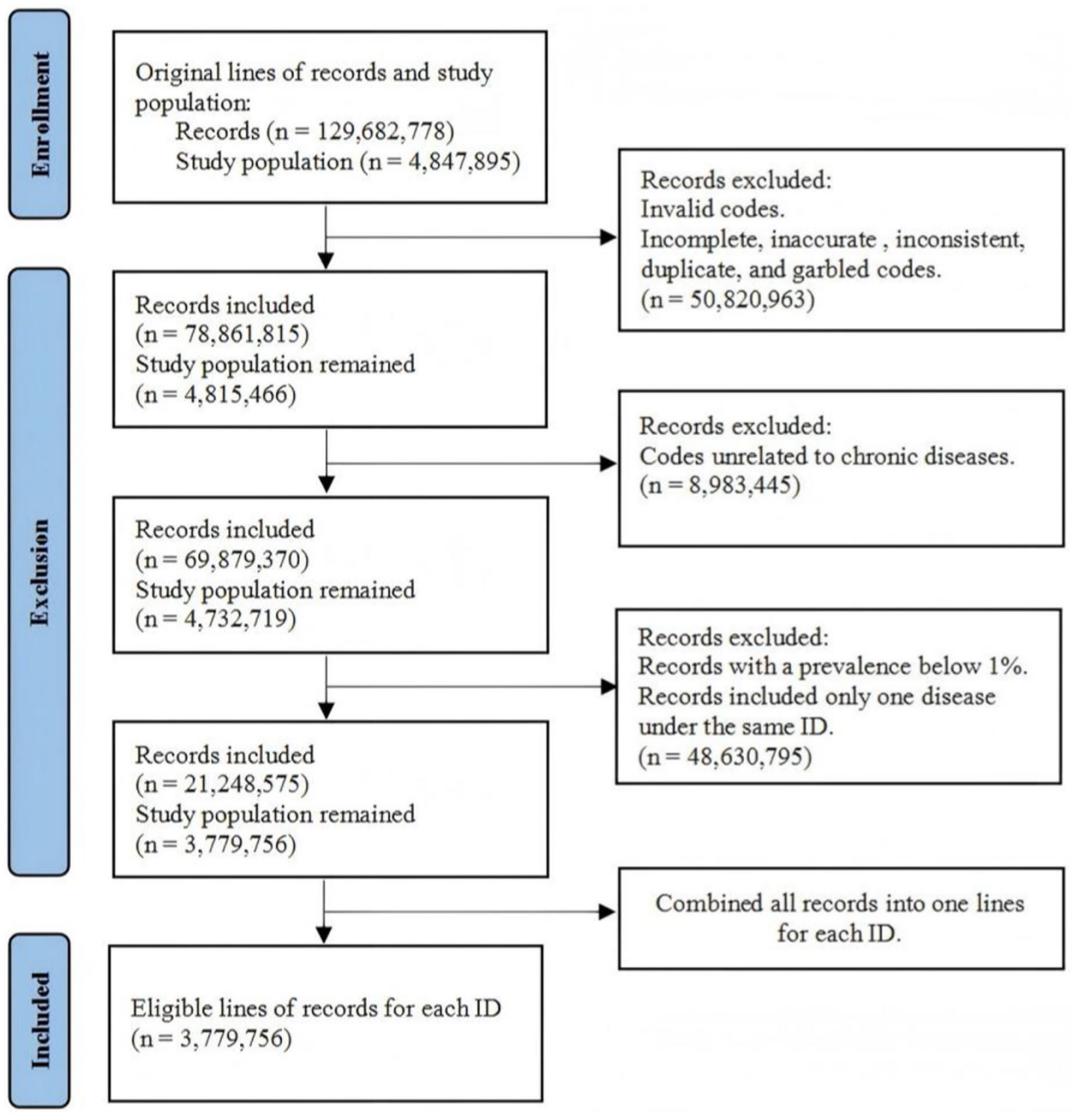
Flowchart of data processing and records exclusion.

All disease codes were referred to ICD-10 (International Classification of Diseases, 10th Revision). Only the records that met ICD-10 coding rules were selected (e.g., A01.001). Incomplete, inaccurate, inconsistent, duplicate, and garbled codes were also directly excluded from the database. Additionally, records that contained only one disease under the same ID were excluded since they were not meaningful for comorbidity analysis. Diseases that were unrelated to chronic diseases were excluded. Diseases with a prevalence below 1% of the total number of records were also excluded. The dataset originally contained 129,682,778 records from 4,847,895 patients aged 60 to 99 years. After processing, this study included 3,779,756 lines of records (1,734,188 for men and 2,045,568 for women) (Figure 1). Then, 150,000 records were randomly sampled by gender for further network analysis and community classification.

### 2.2 Re-coding of diseases

ICD-10 codes contain similar diseases under the same code level, some of which were disordered and included insufficient subjects for analysis. For statistical feasibility, diseases that occur in the same body part with similar pathogenesis were combined to generate a new code. For example, EE03 referred to a combination of E03.8 and E03.9, which represented a sum of hypothyroidism. II2520 referred to a combination of diseases under I25 (chronic ischaemic heart disease) and I20 (angina pectoris) since I20 might be an outcome of I25.

Based on the new coding rules, the top five prevalent diseases were determined first. They were hypertension, ischaemic heart disease (IHD), type 2 diabetes mellitus (T2DM), lipoprotein metabolism disorders (LMD), and gastritis. Then, the top 40 diseases with their respective comorbidity rate of these five diseases were determined. A total number of 38 chronic diseases were retained after deleting duplication (new codes were listed in Table 1). The 38 diseases were hypertension, IHD, T2DM, gastritis, LMD, hypothyroidism, non-toxic diffuse goiter, sleep disorders, sleep apnea, acute myocardial infarction, cardiac arrhythmia, cerebral infarction, cerebrovascular disease, chronic rhinitis, chronic pharyngitis, bronchitis, gastro-esophageal reflux disease with esophagitis (GERD with esophagitis), non-infective gastroenteritis and colitis, constipation, functional diarrhea, polyps of the colon, non-alcoholic fatty liver disease (NAFLD), dermatitis, arthritis, osteoporosis, chronic kidney disease, dizziness and giddiness, anxiety disorders, neurotic disorders, atrial fibrillation and flutter, heart failure, atherosclerosis, chronic obstructive pulmonary disease (COPD), gastric ulcer, hyperuricemia, gonarthrosis, spondylosis, and (peri-)menopausal disorder.

**Table 1:** Results of the centrality of each disease in the comorbidity network of male and female patients.

New code	Disease category	Male			Female		
		Strength	Closeness	Betweenness	Strength	Closeness	Betweenness
i10	Hypertension	2.617	0.005	0	3.882	0.006	14
ii2520*	Chronic IHD**	**10.879**	**0.008**	**83**	**10.327**	**0.008**	**95**
ee1114	T2DM**	3.457	0.006	0	2.880	0.005	0
kk29*	**Gastritis**	**10.178**	**0.007**	**90**	**10.819**	**0.007**	**116**
e78	LMD**	1.285	0.006	0	0	-	0
ee03	Hypothyroidism	1.781	0.005	35	3.446	0.005	34
ee04	Non-toxic diffuse goiter	1.100	0.004	0	1.207	0.004	0
g47_0	Sleep disorders	4.167	0.005	7	3.697	0.005	6
g47_3	Sleep apnea	1.285	0.006	14	0	-	0
i21	Acute myocardial infarction	4.085	0.007	0	2.656	0.007	0
i49	Cardiac arrhythmia	6.842	0.007	23	6.842	0.007	23
i63	Cerebral infarction	4.752	0.007	9	4.606	0.006	9
ii67	Cerebrovascular disease	6.197	0.007	26	5.822	0.007	23
j31_0	Chronic rhinitis	4.494	0.007	39	4.620	0.006	23
j31_2	Chronic pharyngitis	5.495	0.006	21	6.009	0.006	8
jj4042	Bronchitis	5.937	0.007	36	6.442	0.007	42
k21	GERD**with esophagitis	5.219	0.006	0	5.370	0.006	0
k52_9	Non-infective gastroenteritis and colitis	3.423	0.006	11	3.793	0.006	2
k59_0	Constipation	5.219	0.007	13	4.657	0.006	4
k59_1	Functional diarrhea	5.794	0.006	16	5.665	0.006	10
k63_5*	**Polyps of the colon**	**5.846**	**0.007**	**64**	4.713	0.007	37
k76_0*	NAFLD**	**5.918**	**0.007**	**87**	**5.991**	**0.007**	**55**
l30_9	Dermatitis	1.893	0.004	0	2.954	0.004	0
m13	Arthritis	6.480	0.007	24	6.480	0.007	24
m81*	**Osteoporosis**	**7.011**	**0.008**	**70**	6.674	0.007	47
nn18	Chronic kidney disease	7.011	0.007	47	7.146	0.007	37
r42	Dizziness and giddiness	4.238	0.006	19	4.606	0.006	22
f41	Anxiety disorders	4.512	0.006	17	4.449	0.006	18
f48	Neurotic disorders	4.466	0.006	11	3.833	0.005	5
i48	Atrial fibrillation and flutter	5.221	0.007	22	5.628	0.007	21
i50*	**Heart failure**	**9.612**	**0.008**	**97**	**9.671**	**0.008**	**110**
i70*	**Atherosclerosis**	**6.750**	**0.007**	**73**	5.207	0.007	19
j44	Chronic obstructive pulmonary disease	3.804	0.007	33	4.135	0.007	39
k25	Gastric ulcer	2.435	0.006	0	2.245	0.007	0
me1079	Hyperuricemia	3.828	0.007	8	5.000	0.007	10
m17	Gonarthrosis	3.423	0.006	4	3.330	0.006	1
m47	Spondylosis	4.431	0.007	23	4, 431	0.006	23
n95	(peri-)Menopausal disorders	0	-	0	2.919	0.005	1

*Diseases represented in bold text have a strength greater than 5, a closeness equal to or greater than 0.007, and a betweenness greater than 50. These diseases have strong centrality and are easily susceptible to comorbidity. **IHD, ischaemic heart disease; T2DM, type 2 diabetes mellitus; LMD, lipoprotein metabolism disorders; GERD, gastro-esophageal reflux disease; NAFLD, non-alcoholic fatty liver disease.

### 2.3 Statistical analysis

The database was generated using Excel 2013. The characteristics of age were presented as weighted means with a standard deviation (SD). The number of medical visits of the study population by age group and the prevalence of the top five diseases were presented as weighted percentages.

### 2.4 Comorbidity network analysis

Network analysis was conducted using the IsingFit function (*α* = 0.05) with R version 4.3.0. This network estimation employed eLasso, which combines l1-regularized logistic regression with model selection based on the Extended Bayesian Information Criterion (EBIC), enabling the identification of relevant relationships between diseases:


$$P_{\theta }\left(x_{i}=1|x_{\backslash i}\right)=\frac{\exp\left(\sum_{j\varepsilon V\backslash i}w_{ij}x_{j}+b_{i}\right)}{1+\exp\left(\sum_{j\varepsilon V\backslash i}w_{ij}x_{j}+b_{i}\right)}$$

A coefficient *w_ij_* greater than 0 indicates a higher frequency of two diseases occurring simultaneously, suggesting a higher likelihood of comorbidity. On the contrary, a coefficient smaller than 0 indicates a lower frequency of simultaneous occurrence. This is because the appearance of the two diseases in the 0/1 statistical matrix was decentralized, but the association still exists. In this study, positive and negative associations were used to describe the comorbidity frequency. The weight value of edges was used to describe this association in this study.

The network results included diseases as nodes and relevant associations as edges (10, 11). A node represents a single chronic disease. Edge connects two comorbid diseases. The thickness of the edge is proportional to the intensity of comorbidity. A thicker edge indicates a stronger correlation of comorbidity. Strength is defined as the sum of the weights of all nodes. Closeness quantified the indirect distance between two diseases. A greater closeness indicates a shorter distance between two diseases, and they may co-occur more easily. Betweenness indicates the number of times a disease acts as a connecting pathway to other pairs of comorbidity. A higher betweenness emphasizes the importance of the disease and its strong relationship with others (12).

### 2.5 Community classification

The comorbidity network conducted preliminary correlations among the studied diseases. Then, community classification was applied to further examine those positive correlations in depth to focus the results on comorbidity patterns. The analysis of clustering was based on the weights of the edges generated in the network, which introduced a concept of modularity, a quality index. The fast-greedy community function was applied to calculate modularity based on the weights of the edge. Modularity defined as


$$Q=\frac{1}{2m}\sum_{i,j}\left(A_{ij}-\gamma \frac{k_{i}k_{j}}{2m}\right)\delta \left(c_{i},c_{j}\right)$$

where *m* is the number of edges, *A_ij_* is the element of the *A* adjacency matrix in disease *i* and disease *j*. *k_i_* is the sum of weights of adjacent edges for disease *i*. k*j* is the degree of disease *j*. *ci* and *cj* are the components of diseases *i* and *j*.*γ* is a resolution parameter to weight random null model and it is usually set to 1 (13).

### 2.6 Ethics statement

There was no personal information included in the dataset. The Shanghai Medical and Technology Information Institute Ethics Committee approved this study and the use of the dataset (No.2024004). All participants and procedures followed the required guidelines (14).

## 3 Results

### 3.1 Characteristics of the study population

This study initially analyzed the distribution of the study population based on their number of visits regarding single-diagnosed diseases across different age groups, stratified by gender. The overall percentage of medical visits of patients aged 60 to 69 years was 47.28% (men: 22.26%, women: 25.02%). The percentages for age groups 70–79, 80–89, and 90–99 were 33.99% (men: 16.04%, women: 17.95%), 14.37%, (men: 6.13%, women: 8.25%) and 4.36% (men: 1.46%, women: 2.90%), respectively. The average age for the study population was 72.87 (SD, 8.578) years. 72.52 (SD, 8.320) for men and 73.13 (SD, 8.758) for women (Table 2).

**Table 2:** Basic characteristics of the study population.

Characteristics	Total		Male		Female	
Age (year)	No. of visits*	%	No. of visits	%	No. of visits	%
60–69	1,787,022	47.28	841,369	22.26	945,653	25.02
70–79	1,284,756	33.99	606,310	16.04	678,446	17.95
80–89	543,220	14.37	231,466	6.13	311,754	8.25
90–99	164,758	4.36	55,043	1.46	109,715	2.90
Age (year)	Average	SD	Average	SD	Average	SD
60–99	72.87	8.578	72.52	8.320	73.13	8.758

*The number of visits represents the prevalence of each disease. There was no duplication of diagnosis in this study.

### 3.2 Top 10 most visiting diseases

Hypertension had the highest frequency of visits among all the study subjects, with a total of 11,422,288 visits for men and 1,306,260 visits for women. The top 10 diseases were hypertension, IHD, T2DM, LMD, gastritis, sleep disorders, conjunctivitis, intestinal disorders, bronchitis, and respiratory disorders (Table 3).

**Table 3:** Top 10 single-diagnosed diseases based on the number of medical visits of the study population.

Top 10 single-diagnosed diseases	Total No. Visits (%*)	Male	Female
1. Hypertension	2,448,548 (64.78)	1,142,288	1,306,260
2. IHD**	1,476,396 (39.06)	637,315	839,081
3. T2DM**	943,812 (24.97)	465,184	478,628
4. LMD**	823,572 (21.79)	329,847	493,725
5. Gastritis	744,880 (19.71)	311,084	433,796
6. Sleep disorders	667,676 (17.66)	272,915	394,761
7. Conjunctivitis	518,629 (13.72)	195,735	322,894
8. Intestinal disorders	513,921 (13.60)	218,230	295,691
9. Bronchitis	458,076 (12.12)	199,803	258,273
10. Respiratory disorders	430,433 (11.39)	200,616	229,817

*Prevalence of each disease based on the total study population (3,779,756). **Explanation of abbreviation: IHD-ischaemic heart disease; T2DM-type 2 diabetes mellitus; LMD-lipoprotein metabolism disorders.

### 3.3 The comorbidity network of the top five diseases within all 38 analyzed diseases

**Figure 2:**
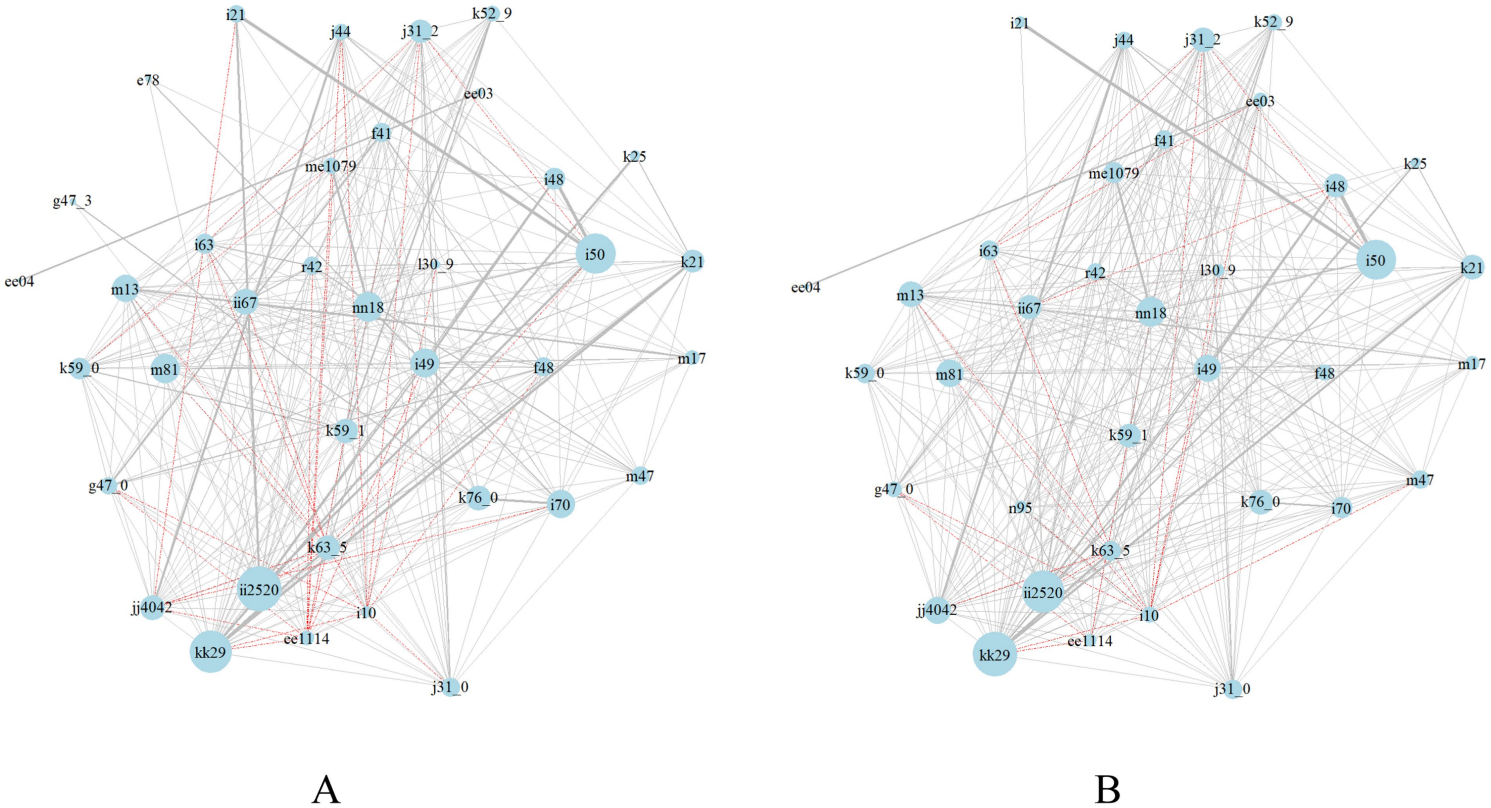
Comorbidity network in men **(A)** and women **(B)** aged 60 to 99 years. Nodes represent chronic diseases. Node size is proportional to the strength of diseases. Edges connect comorbid diseases. Black edges indicate positive correlations and red edge indicates negative correlations. Edge thickness indicates the intensity of the correlation.

Both comorbidity networks for men and women, as illustrated in Figure 2, indicated that hypertension positively correlated with 11 diseases. These included IHD, hyperuricemia, chronic kidney failure, cerebral infarction, T2DM, cerebrovascular disease, arthritis, cardiac arrhythmia, dizziness and giddiness, bronchitis, and NAFLD (weight of edges greater than 0, see Supplementary Tables 1, 2). Conversely, hypertension exhibited a negative correlation with chronic pharyngitis, gastritis, polyps of the colon, sleep disorders, and dermatitis (weight of edges lower than 0, see Supplementary Tables 1, 2). There was an additional negative association with neurotic disorder, chronic rhinitis, and COPD in men than in women. These diseases had a lower frequency of occurring simultaneously due to the decentralization of the records. Hypertension had an additional positive correlation between atrial fibrillation and flutter, constipation, and gonarthrosis, and an additional negative correlation with osteoporosis, spondylosis, hypothyroidism, and (peri-)menopausal disorders.

T2DM was found to be positively associated with nine diseases and negatively associated with three diseases. The nine diseases were chronic kidney failure, NAFLD, hypertension, atherosclerosis, heart failure, IHD, cerebral infarction, osteoporosis, and constipation. The three diseases were chronic pharyngitis, sleep disorders, and gastritis. Additionally, men were more likely than women to have acute myocardial infarction and less likely to have dizziness and giddiness, polyps of the colon, bronchitis, hyperuricemia, and COPD alongside T2DM. In women, T2DM additionally exhibited positive associations with hyperuricemia, arthritis, bronchitis, gastroenteritis and colitis, and dermatitis.

There were positive correlations between IHD and 28 diseases which included cardiovascular pathology (e.g., acute myocardial infarction, heart failure, cardiac arrhythmia, atrial fibrillation and flutter, cerebrovascular disease, hypertension, cerebral infarction, atherosclerosis, and LMD), chronic kidney failure, osteoporosis, arthritis, bronchitis, chronic pharyngitis, sleep disorders, T2DM, functional diarrhea, NAFLD, chronic rhinitis, gonarthrosis, and gastroenteritis and colitis. Women were additionally found to have hyperuricemia, gastric ulcer, spondylosis, and dermatitis with IHD.

Gastritis was positively associated with gastrointestinal (GI) disorders such as GERD with esophagitis, gastric ulcers, polyps of the colon, gastroenteritis and colitis, functional diarrhea, and constipation. It was also positively correlated with 26 other diseases, such as chronic pharyngitis, chronic rhinitis, NAFLD, anxiety disorders, bronchitis, osteoporosis, cardiac arrhythmia, IHD, sleep disorders, and COPD. Furthermore, it was negatively correlated with T2DM and hypertension. As mentioned above, the negative correlation indicated a lower frequency of existing simultaneously. The comorbidity pairs still existed. Women additionally had cerebral infarction, hyperuricemia, and (peri-) menopausal disorders positively associated with gastritis.

In men, LMD was positively associated with atherosclerosis, IHD, and hyperuricemia. It should be noted that women were not sampled for LMD and its associated diseases due to the low frequency of comorbidity (Figure 2).

### 3.4 Network centrality of the 38 analyzed diseases

This study identified diseases with a strength greater than 5, a closeness equal to or greater than 0.007, and a betweenness greater than 50 as having strong centrality and were easily susceptible to comorbidity. Regarding the top five prevalent diseases, IHD (men: s = 10.879, c = 0.008, b = 83; women: s = 10.327, c = 0.008, b = 95) and gastritis (men: s = 10.178, c = 0.007, b = 90; women: s = 10.819, c = 0.007, b = 116) were the major two diseases that would easily be susceptible to comorbidity in both men and women. Among the 38 diseases, NAFLD (men: s = 5.918, c = 0.007, b = 87; women: s = 5.991, c = 0.007, b = 55), polyps of the colon (men: s = 5.846, c = 0.007, b = 64), osteoporosis (men: s = 7.011, c = 0.008, b = 70), and atherosclerosis (men: s = 6.750, c = 0.007, b = 73) were strongly associated with other diseases. Furthermore, the centrality of heart failure (men: s = 9.612, c = 0.008, b = 97; women: s = 9.671, c = 0.008, b = 110) was strong, indicating that this disease could commonly develop into outcome complications and thus correlated with the majority of the analyzed chronic diseases (Table 1).

### 3.5 Network-based community classification of the top five prevalent diseases

**Figure 3:**
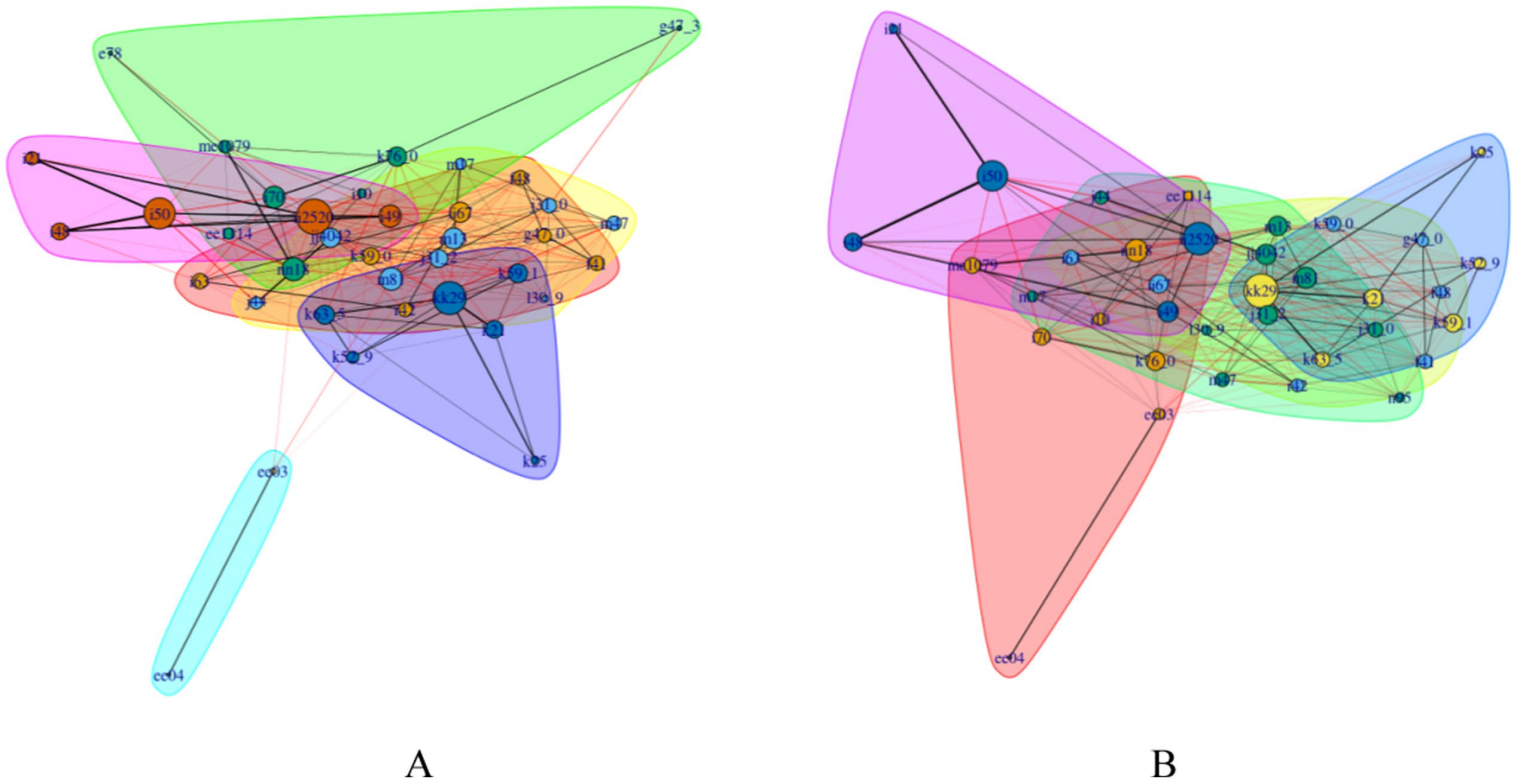
Network-based community clustering in men **(A)** and women **(B)** aged 60 to 99 years. Definitions of node, node size, and edge thickness remain constant, as described in Figure 2. Each cluster with a specific color refers to a disease community. Edges within the same cluster are in black. Edges across different clusters are in red. The color of nodes remains the same within the cluster.

Modularities for clustering were both greater than 0.3 by gender (men: 0.34, women: 0.31), indicating that this method is effective in clearly categorizing the target diseases into smaller communities. In men, a cluster was observed among T2DM, hypertension, chronic kidney failure, hyperuricemia, NAFLD, atherosclerosis, LMD, and sleep apnea. A cluster was identified among IHD, cardiac arrhythmia, heart failure, acute myocardial infarction, and atrial fibrillation and flutter. Gastritis clustered with polyps of the colon, gastroenteritis and colitis, GERD with esophagitis, and gastric ulcers (Figure 3A). In women, a cluster was observed among T2DM, chronic kidney failure, hyperuricemia, hypothyroidism, NAFLD, atherosclerosis, non-toxic diffuse goiter, and hypertension. Gastritis clustered with polyps of the colon, gastritis, GERD with esophagitis, functional diarrhea, and gastric ulcers. IHD clustered with cardiac arrhythmias, atrial fibrillation flutter, heart failure, and acute myocardial infarction (Figure 3B).

Additional clusters were identified. In men, sleep disorders were clustered with cerebral infarction, cerebrovascular disease, dizziness and giddiness, neurotic disorders, constipation, and anxiety disorders. Bronchitis was associated with arthritis, dermatitis, COPD, chronic rhinitis and pharyngitis, osteoporosis, gonarthrosis, and spondylosis. Hypothyroidism was associated with non-toxic diffuse goiter. In women, sleep disorders were clustered with constipation, cerebral infarction, cerebrovascular disease, dizziness and giddiness, anxiety disorders, and neurotic disorders. Bronchitis was clustered with osteoporosis, arthritis, dermatitis, chronic rhinitis and pharyngitis, osteoarthrosis, gonarthrosis, spondylosis, (peri-)menopausal disorders, and COPD (Figure 3).

## 4 Discussion

### 4.1 Chronic ischemic heart disease and its comorbidity patterns

**IHD** is associated with chronic diseases such as cardiovascular pathology, GI disorders, chronic kidney failure, osteoporosis, arthritis, bronchitis, chronic pharyngitis, sleep disorders, T2DM, NAFLD, chronic rhinitis, and gonarthrosis. IHD is one of the major types of cardiovascular disease (CVD) developing in China. Known studies have analyzed that IHD is closely associated with hypertension, T2DM, LMD, overweight or obesity, and unhealthy lifestyles. Risk factors are not significantly different between men and women (15). This study indicated that IHD is associated with not only commonly recognized CVD, cerebrovascular diseases, and T2DM but also abnormalities of hepatic and renal function, upper respiratory problems, lung diseases, bone and joint diseases, mental disorders, and GI disorders, which are susceptible to comorbidity in older patients with IHD. Deterioration of renal function is one of the most common comorbidities of IHD and CVD, which is often accompanied by heart failure as well (16, 17). NAFLD and CVD are manifestations of end-organ damage in the metabolic syndrome, and they are associated with each other through multiple mechanisms. Patients with IHD are likely to have atherosclerosis, cardiomyopathy, and arrhythmia, which contribute to CVD morbidity and mortality (18). CVD and IHD are also the most common comorbidities of COPD, accelerating disease progression, increasing risk factors, and resulting in the usage of therapeutic agents (19). Gastritis, gastric ulcers, osteoarthritis, and upper respiratory tract problems, such as chronic nasopharyngitis and bronchitis, induced by IHD are triggered by inflammatory mechanisms, a very common comorbidity pattern in older patients (20). Mental disorders such as sleep disorders, anxiety disorders, and vertigo are triggered by stress (21). This pattern is associated with a long duration of illness, high therapeutic difficulty, and high mortality of IHD. In addition, the comorbidity of hyperuricemia, dermatitis, and spondylosis in women with IHD was identified. Although asymptomatic hyperuricemia has long been considered to be a marker of metabolic disorders, several prospective large-scale clinical studies suggested that it may be an independent risk factor for CVD and IHD, and is strongly associated with poor outcomes of cardiac, vascular, and renal problems (22, 23).

### 4.2 Early detection and implications for disease control for chronic ischemic heart disease comorbidity patterns

Due to the complexity of IHD comorbidity patterns in older patients, it is necessary to apply a multidisciplinary diagnosis strategy to monitor these conditions in the early stage. It is crucial to regularly screen blood pressure, blood glucose, serum lipid level, hepatic and renal function, cardio-respiratory function, inflammatory response, and other physiological conditions for IHD patients (24–26). The intervention strategy should focus on preventing end-organ damages associated with metabolic syndrome, such as atherosclerosis, myocardial problems, and arrhythmias. Due to the strong association between IHD, heart failure, CVD, COPD, and decline in renal function, it is therefore recommended that monitoring cardiac, pulmonary, and renal functions plays an important role in preventing the potential morbidity of IHD (16). NAFLD and IHD interact through similar metabolic pathways. We recommended that IHD patients ease the double burden of the liver and cardiovascular system by weight management and glycaemic control. Women with IHD are more likely to have hyperuricemia, dermatitis, and spondylosis problems. It will be helpful to monitor inflammatory levels of the whole body to prevent adverse complications (27).

### 4.3 Chronic gastritis and its comorbidity patterns

Although the results of the community classification demonstrated that gastritis comorbidity patterns in older patients were predominantly associated with conventional GI diseases, network analysis provided a more general vision of all the potential diseases that are susceptible to gastritis. The comorbidity of gastritis with upper respiratory tract diseases may be attributed to acid reflux, which irritates the mucosa of the digestive system and ultimately exacerbates symptoms (28). A long-term diet high in fat and cholesterol may impact hepatic metabolism, potentially leading to the development of NAFLD. This is followed by the presentation of impaired endocrine function, which reduces the decomposition ability of greasy food, and then elevated GI burden (29). The prevalence of gastritis, ulcerative esophagitis, duodenitis, and other GI diseases is increased in patients with chronic kidney failure and hyperuricemia (30). Patients with gastritis are also susceptible to mental disorders, which result from the psychological burden due to long-term treatment (31). The comorbidity pattern between gastritis and hypothyroidism is also significant in older patients because hypothyroidism may lower appetite and slow down GI peristalsis. In this case, gastric disorders such as gastritis, dyspepsia, and constipation may happen (32, 33). Additionally, chronic gastritis may result in a systemic low-grade inflammatory response, thereby increasing the risk of vascular endothelial damage and CVD (34). It is important to highlight the increased prevalence of menopausal disorders, cerebral infarction, and hyperuricemia in women with gastritis compared to men. During the menopausal stage, as the decline of ovarian function lowers estrogen levels, it leads to endocrine disruption and results in changes in hormone levels, which may affect GI tract function, thereby increasing the risk of gastritis (35). Hormone fluctuation affects gut microbiota dysbiosis in the menopausal stage, which can also affect GI functions (36). In addition, cerebral infarction and hyperuricemia are risks resulting from the long-term use of non-steroidal anti-inflammatory drugs (NSAIDs) and unhealthy diets.

### 4.4 Early detection and implications for disease control for gastritis comorbidity patterns

Most patients with chronic gastritis and its morbidity have been taking anti-inflammatory agents and other medications for a long period. Consequently, the primary objective of risk prevention should be protecting GI mucosa by selecting appropriate medications (37). It is recommended that patients should take medications with less irritation to GI mucosa, along with mucosal protectors and slow-released preparations, or take medicine after meals (38). The side effects of medications should be regularly assessed. It is recommended that older patients should intake low-fat, low-cholesterol diets to promote GI function. GI symptoms such as dyspepsia and constipation in gastritis patients with hypothyroidism need to be monitored frequently to facilitate timely intervention. Additionally, chronic gastritis can also impose a systemic low-grade inflammatory response, thereby increasing the risk of CVD (34). In treating complications, the usage of medications with multi-target effects is a priority, along with appropriate adjustment of quantity, dosage, and frequency to reduce side effects. Furthermore, women with gastritis around 60 years should be particularly concerned about the potential comorbidities associated with menopausal diseases, such as cerebral infarction and hyperuricemia. Given the fluctuations in hormone levels during this phase of the disease, it is important to monitor endocrine function frequently, as it may impact GI conditions (39).

### 4.5 A ternary comorbidity pattern of type 2 diabetes mellitus, hypertension, and lipid metabolism disorders

T2DM, hypertension, and LMD were identified in the same community with some other chronic diseases such as hyperuricemia and hypothyroidism for women and sleep apnea for men, which indicated that these chronic diseases were closely related, and hypertension is prone to comorbid with metabolic syndromes (40). The major causes of this comorbidity pattern include insulin resistance, chronic inflammatory response, obesity, genetic factors, endothelial dysfunction, unhealthy lifestyle, and renal impairment. These factors interact to form a complex network of metabolic syndromes. The coexistence of chronic renal failure and hyperuricemia in this pattern may result from the fact that insulin resistance elevated uric acid levels (41, 42). Chronic inflammatory response exacerbates insulin resistance by triggering responses of C-reactive protein (CRP), tumor necrosis factor-alpha (TNF-*α*), and interleukin-6 (IL-6), resulting in T2DM and renal impairment and increasing uric acid levels (43, 44). Meanwhile, elevated blood glucose in the liver activates glycogen synthase to accumulate hepatic fat, leading to LMD an**d** NAFLD (45). In women, the decreasing insulin sensitivity is associated with the decreased metabolic rate of thyroid hormones, which triggers hypothyroidism and goiter (46). Both T2DM and hypothyroidism increase the risk of high cholesterol and hypertension, which are common cardiovascular risk factors in people with T2DM. Sleep apnea syndrome in male patients will lead to frequent apneic episodes and hypoxemia, which is associated with hypertension by activating and raising oxidation (47). Activating the sympathetic nervous system may contribute to insulin resistance, which can increase the risk of T2DM (48).

### 4.6 Early detection and implications for disease control for comorbidity pattern of type 2 diabetes mellitus, hypertension, and lipid metabolism disorders

The prevention of this ternary comorbidity pattern should focus on regulating blood glucose and insulin levels by pharmacological approaches such as in-taking metformin and insulin sensitizers. It is recommended that a healthy lifestyle, including weight management, frequent physical activity, and low-sugar, low-fat diets, can improve insulin sensitivity, overall metabolic status, and lower inflammation makers (49, 50). Regular detections of hepatic and renal function can help identify early impairment in older patients with T2DM and hyperuricemia.

It is also essential to consider gender factors in this comorbidity pattern. Female patients are recommended to regularly assess thyroid gland function and hormone levels due to the potential interaction between hypothyroidism and T2DM. Male patients undergoing sleep apnea syndrome should specifically screen their blood pressure and blood glucose. Using a respiratory assistant device and modifying sleeping posture to improve sleep quality. Taking less fats, sugar, salts, and alcohol will reduce the progression of comorbidity.

### 4.7 Mental health management and patient self-management

Neurotic disorders, sleep disorders, and other psychiatric factors were prevalent in the comorbidity pattern discussed above. It can be inferred that the long-term treatment of comorbidity in older adults is often accompanied by challenges on the psychological burden, such as anxiety and depression, which have a significant impact on patients’ life quality and motivation for treatment. Comorbidities, such as insomnia and neurotic disorders, will develop into a chronic inflammatory response that affects cardiovascular, cerebral, respiratory, and GI health. Therefore, strengthening emotional support for older patients with comorbidity is an indispensable part of their treatment. This not only helps to reshape their confidence but also significantly improves their cooperation and ensures the effectiveness of treatment plans. For patients presenting with severe emotional disturbances, it is recommended that close collaboration with psychologists be established to implement psychological interventions and emotional relief strategies. Furthermore, self-management plays an active role in maintaining health conditions. Patients are encouraged to learn basic disease mechanisms and how to utilize medical tools to regularly monitor their health data, such as heart rate, blood pressure, blood glucose, and uric acid, to take immediate action to any potential health problems.

### 4.8 Implications and potential policy actions

Shanghai is a typical region in China facing comorbidity challenges. The network analysis is an effective approach for public health practitioners to quickly identify potential comorbidity patterns among the target population and then take action to reduce risk factors of disease development. This study discovered pre-existing comorbidity patterns and unusual ones, providing evidence to strengthen more effective management strategies. New-identified patterns may draw more attention from practitioners for the development of novel technology and strategies to monitor and prevent risk factors. Currently, China is implementing community-based primary medical services. It offers free screening for common chronic diseases such as T2DM, ICH, and GI diseases. Patients with T2DM could have additional screenings on thyroid function, uric acid levels, and sleeping quality. In addition, residents are encouraged to enroll in the family doctor program. Family doctors are suggested to provide personalized medical consultations regarding the potential comorbidity patterns older adults may have, helping them strengthen comorbidity monitoring, arrange appropriate follow-up visits, and maintain good mental health. These actions aim to prevent and slow down the development of comorbidity and reduce any further medical burden for both patients and the public health system.

### 4.9 Limitations

The dataset was derived from cross-sectional medical records, so it is not appropriate to conclude causality. There were no covariates, such as educational level, smoking, and drinking alcohol, including for data analysis because medical records did not contain such information. In addition, some diseases were initially categorized vigorously in ICD-10. In this case, the final subjects included in some disease groups might be less than or more than expected, which leads to bias in the analysis. Moreover, the results only represented some general comorbidity patterns in the region of Shanghai, which lacked normality across the whole target population in China. Therefore, the implications for disease control could only be applied within the specific region.

## 5 Conclusion

The visualized analysis revealed that the most two prevalent comorbidity patterns among the study population were dominated by IHD and gastritis, followed by a ternary pattern of hypertension, T2DM, and LMD. Men are more likely to simultaneously have cardiovascular and sleep problems than women when they suffer from the top five prevalent diseases, whereas thyroid disease, chronic inflammatory diseases, and hyperuricemia are more commonly comorbid within women. By monitoring vital signs, such as heart rate, blood pressure, blood glucose, hepatic and renal function, and inflammatory markers, and their influencing factors within comorbidity patterns, healthcare professionals may take immediate actions toward the potential onset of comorbidity. Furthermore, it is vital to pay more attention to the mental health of patients who suffer from long-term chronic diseases and undergo complex treatment over a long period. Given the accelerating aging problem in Shanghai, it is insufficient to intervene after diseases occur. Early detection and prevention strategies are also necessary to control the onset, progression, and mortality rates. This study provided a general insight into the current comorbidity situation among older adults in Shanghai and suggestions on prevention strategies, which may help control the prevalence of chronic diseases.

## Data Availability

The data analyzed in this study is subject to the following licenses/restrictions: Dataset is not available to the public due to ethical restrictions. Requests to access these datasets should be directed to niuyuhong@126.com.
