# Supplementary material for: Comorbidity patterns and implications for disease control: a network analysis of medical records from Shanghai, China
Source: Front Public Health. 2025 Mar 10;13:1516215. doi: 10.3389/fpubh.2025.1516215 (PMC11930827; doi:10.3389/fpubh.2025.1516215)
Supplement: Supplementary file 1 [file Table_1.docx]

Supplementary table 1：the weight of edges of network analysis for males

|  | Hypertension | T2DM | IHD | Gastritis | LMD |
| --- | --- | --- | --- | --- | --- |
| IHD | 0.432 | 0.216 | 0 | 0.204 | 0.418 |
| Hyperuricaemia | 0.372 | -0.177 | 0 | 0 | 0.147 |
| Chronic kidney disease | 0.347 | 0.565 | 0.414 | 0.157 | 0 |
| Cerebral infarction | 0.332 | 0.214 | 0.404 | 0 | 0 |
| T2DM | 0.297 | 0 | 0.216 | -0.097 | 0 |
| Cerebrovascular disease | 0.085 | 0 | 0.510 | 0.160 | 0 |
| Arthritis | 0.066 | 0 | 0.288 | 0.290 | 0 |
| Cardiac arrhythmia | 0.050 | -0.056 | 1.021 | 0.215 | 0 |
| Dizziness and giddiness | 0.037 | -0.047 | 0.127 | 0.190 | 0 |
| NALFD | 0.031 | 0.411 | 0.165 | 0.315 | 0 |
| Bronchitis | 0.007 | -0.075 | 0.245 | 0.292 | 0 |
| GERD with oesophagitis | 0 | 0 | 0.106 | 1.601 | 0 |
| Gastric ulcer | 0 | 0 | 0 | 1.316 | 0 |
| Noninfective gastroenteritis and colitis | 0 | 0 | 0.035 | 0.638 | 0 |
| Functional diarrhoea | 0 | 0 | 0.226 | 0.616 | 0 |
| Anxiety disorders | 0 | 0 | 0.216 | 0.297 | 0 |
| Osteoporosis | 0 | 0.147 | 0.391 | 0.290 | 0 |
| Constipation | 0 | 0.064 | 0.318 | 0.277 | 0 |
| Spondylosis | 0 | 0 | 0 | 0.261 | 0 |
| Gonarthrosis | 0 | 0 | 0.036 | 0.127 | 0 |
| Hypothyroidism | 0 | 0 | 0 | 0.082 | 0 |
| Atherosclerosis | 0 | 0.287 | 0.372 | 0.056 | 0.720 |
| Acute myocardial infarction | 0 | 0.133 | 1.589 | 0 | 0 |
| Heart failure | 0 | 0.242 | 1.323 | 0 | 0 |
| Atrial fibrillation and flutter | 0 | 0 | 0.767 | 0 | 0 |
| LMD | 0 | 0 | 0.418 | 0 | 0 |
| (peri-)Menopausal disorders | 0 | 0 | 0 | 0 | 0 |
| Sleep apnoea | 0 | 0 | 0 | 0 | 0 |
| Nontoxic diffuse goitre | 0 | 0 | 0 | 0 | 0 |
| Hypertension | 0 | 0.297 | 0.432 | -0.061 | 0 |
| Neurotic disorders | -0.025 | 0 | 0.245 | 0.216 | 0 |
| Chronic pharyngitis | -0.037 | -0.050 | 0.192 | 0.381 | 0 |
| Chronic rhinitis | -0.051 | 0 | 0.081 | 0.385 | 0 |
| Gastritis | -0.061 | -0.097 | 0.204 | 0 | 0 |
| Polyp of colon | -0.073 | -0.073 | -0.260 | 1.298 | 0 |
| Sleep disorders | -0.090 | -0.070 | 0.173 | 0.179 | 0 |
| Dermatitis | -0.094 | 0 | 0 | 0.126 | 0 |
| Chronic obstructive pulmonary disease | -0.131 | -0.238 | 0.106 | 0.053 | 0 |

Supplementary table 2：the weight of edges of network analysis for females

|  | Hypertension | T2DM | IHD | Gastritis | LMD |
| --- | --- | --- | --- | --- | --- |
| T2DM | 0.592 | 0 | 0.229 | -0.035 | 0 |
| IHD | 0.557 | 0.229 | 0 | 0.267 | 0 |
| Hyperuricaemia | 0.522 | 0.199 | 0.190 | 0.068 | 0 |
| Cerebral infarction | 0.455 | 0.181 | 0.490 | 0.077 | 0 |
| Chronic kidney disease | 0.389 | 0.508 | 0.482 | 0.177 | 0 |
| Cerebrovascular disease | 0.136 | 0 | 0.557 | 0.197 | 0 |
| Arthritis | 0.117 | 0.076 | 0.349 | 0.254 | 0 |
| Bronchitis | 0.107 | 0.046 | 0.321 | 0.304 | 0 |
| NAFLD | 0.069 | 0.423 | 0.179 | 0.345 | 0 |
| Atrial fibrillation and flutter | 0.063 | 0 | 0.809 | 0 | 0 |
| Dizziness and giddiness | 0.061 | 0 | 0.134 | 0.198 | 0 |
| Constipation | 0.046 | 0.067 | 0.335 | 0.294 | 0 |
| Cardiac arrhythmia | 0.046 | 0 | 1.035 | 0.208 | 0 |
| Gonarthrosis | 0.029 | 0 | 0.096 | 0.110 | 0 |
| GERD with oesophagitis | 0 | 0 | 0.106 | 1.544 | 0 |
| Gastric ulcer | 0 | 0 | 0.054 | 1.158 | 0 |
| Noninfective gastroenteritis and colitis | 0 | 0.039 | 0.083 | 0.633 | 0 |
| Functional diarrhoea | 0 | 0 | 0.247 | 0.573 | 0 |
| Chronic rhinitis | 0 | 0 | 0.071 | 0.414 | 0 |
| Anxiety disorders | 0 | 0 | 0.163 | 0.291 | 0 |
| Neurotic disorders | 0 | 0 | 0.259 | 0.260 | 0 |
| Atherosclerosis | 0 | 0.124 | 0.316 | 0.116 | 0 |
| Chronic obstructive pulmonary disease | 0 | 0 | 0.275 | 0.113 | 0 |
| Heart failure | 0 | 0.222 | 1.157 | 0 | 0 |
| Acute myocardial infarction | 0 | 0 | 0.591 | 0 | 0 |
| LMD | 0 | 0 | 0 | 0 | 0 |
| Sleep apnoea | 0 | 0 | 0 | 0 | 0 |
| Nontoxic diffuse goitre | 0 | 0 | 0 | 0 | 0 |
| Hypertension | 0 | 0.592 | 0.557 | -0.044 | 0 |
| Osteoporosis | -0.025 | 0.071 | 0.306 | 0.266 | 0 |
| Chronic pharyngitis | -0.044 | -0.019 | 0.229 | 0.356 | 0 |
| Gastritis | -0.044 | -0.035 | 0.267 | 0 | 0 |
| Dermatitis | -0.052 | 0.022 | 0.016 | 0.122 | 0 |
| Polyp of colon | -0.065 | 0 | -0.187 | 1.414 | 0 |
| Spondylosis | -0.065 | 0 | 0.021 | 0.268 | 0 |
| Sleep disorders | -0.076 | -0.028 | 0.216 | 0.208 | 0 |
| Hypothyroidism | -0.112 | 0 | 0 | 0.129 | 0 |
| (peri-)Menopausaldisorder | -0.209 | 0 | 0 | 0.375 | 0 |
